# EDDM3A drives gastric cancer progression by promoting HIF-1α-dependent aerobic glycolysis

**DOI:** 10.1038/s41389-022-00379-6

**Published:** 2022-01-17

**Authors:** Tao Wu, Ying Yang, Bo Zhang, Zhan-sheng Zhang, Shuai Zhou, Guo-zhan Jia, Shi-qi Liu, Xian-li He, Jia-xing He, Nan Wang

**Affiliations:** Department of General Surgery, Tangdu Hospital, The Air Force Medical University, 710038 Xi’an, China

**Keywords:** Gastric cancer, Oncogenes

## Abstract

Epididymal protein 3A (EDDM3A) is a protein involved in sperm maturation. It has been demonstrated that EDDM3A expression is upregulated and promotes cell proliferation in non-small cell lung cancer (NSCLC). However, the role of EDDM3A in other types of human cancers, including gastric cancer (GC), is still unexplored. Here, we show that the expression of EDDM3A is significantly upregulated in gastric cancer (GC) tissues and its upregulation correlates with poorer survival in patients with gastric cancer. Knockdown of EDDM3A inhibited growth and metastasis of GC cells, whereas overexpression of EDDM3A exhibited the opposite effect. Mechanistically, enhanced aerobic glycolysis mediated by upregulation of HIF-1α and subsequently increased target glycolytic genes and decreased mitochondrial biogenesis was found to contribute to the promotion of tumor growth and metastasis by EDDM3A in GC cells. Additionally, upregulation of EDDM3A in GC is at least partially mediated by downregulation of miR-618. In conclusion, elevated EDDM3A plays a pivotal oncogenic role in gastric carcinogenesis, suggesting it as a potential therapeutic target for treatment of GC.

## Graphical abstract

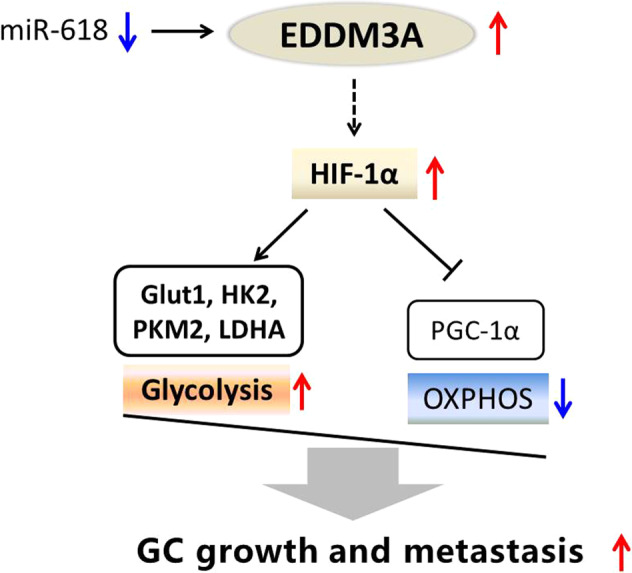

## Introduction

Gastric cancer (GC) is one of the fifth most common malignancies and the third leading cause of cancer-related mortality worldwide. Almost half of all global GC cases are diagnosed in Eastern Asia [1, 2]. Despite several advances in the management of GC, the effective therapies for GC and long-term survival of patients remain poor, mainly due to the poor understanding of novel causative molecular leading to malignant transformation [3, 4]. Therefore, it is extremely urgent to further explore the molecular mechanisms that regulate GC and to develop the novel therapies.

Epididymal protein 3A (EDDM3A) gene is located on human chromosome (chr) 14q11.2, which encodes a protein involved in the maturation of sperm [5]. A previous study in non-small cell lung cancer (NSCLC) has revealed that EDDM3A expression is upregulated in NSCLC tumor tissues as compared with matched adjacent non-tumor tissues and promotes cell proliferation of NSCLC [6]. However, the role of EDDM3A in other types of human cancers, especially the underlying mechanism still unexplored, including gastric cancer (GC).

Our bioinformatics analysis using UALCAN online tool indicated that EDDM3A is markedly upregulated in GC. In the present study, we explored the expression pattern, clinical significance and biological functions of EDDM3A in gastric cancer.

## Materials and methods

### Cell culture

Human gastric cancer cell lines BGC-823, SGC-7901, AGS, MGC-803, MKN-45, GSS, and normal gastric mucosal epithelial cell line GES-1 were maintained in RPMI1640 medium (Invitrogen) with 10% fetal bovine serum (FBS), penicillin (100 IU/mL) and streptomycin (100 μg/mL) at 37 °C with 5% CO_2_. Each cell line was have been authenticated by short tandem repeat (STR) method.

### Vector construction and cell transfection

To knockdown EDDM3A in GC cells, small interfering RNA (Genepharma) corresponding to EDDM3A (Target sequence: 5′-GAGAGAATTCATAAAACTTCATT-3′) was used. To construct small hairpin RNA (shRNA) expression vector corresponding to EDDM3A, a pSilencer 2.1-U6 puro vector (Thermo Fisher Scientific) was used. Stable transfection in BGC-823 cells was selected with puromycin (Thermo Fisher Scientific) within 2 months. To overexpress EDDM3A, the full-length cDNA of EDDM3A, including 3′UTR, was amplified and constructed into a pCDNA-3.1 vector. Lipofectamine 3000 reagent (Invitrogen) was used for the transfections of both siRNAs and plasmids following the manufacturer’s instructions. Small hairpin RNA (shRNA) was constructed using pSilencer 2.1-U6 puro vector to knockdown CD151 expression.

### Tissues collection and IHC staining

A total of 414-paired primary GC tumor tissues (T) and adjacent non-tumor tissues (N) were collected from patients who undergone surgery at the second affiliated hospital of the Air Force Medical University (Xi’an, China) from January 2009 to December 2012 with written informed consent from all patients. Among them, thirty paired samples of GC tumor tissues and adjacent non-tumor tissues were used for qRT-PCR analysis, and the other 384 paired samples were used for IHC staining. All procedures were in accordance with the Ethics Committee of the Air Force Medical University and with the Helsinki Declaration of 1964 and later versions. Informed consent was obtained from all patients.

For IHC staining, sections were incubated with specific primary antibodies listed in the Supplementary Table 1 (Table S1). IHC staining intensity was scored by two independent pathologists based on the intensity and percentage of positive staining.

### qRT-PCR

Total RNA were extracted from GC cells using TRIzol reagent (Invitrogen), and then transcribed into cDNA. SYBR Green reagents were utilized for quantitative real-time PCR (qRT-PCR) with primers provided in Table S2. The β-actin gene was used as internal control.

### Western blot

For total protein extraction, GC cells were lysed with lysis buffer supplemented with protease inhibitor cocktail (Roche) on ice for 20 min. Proteins were electrophoresed and separated on SDS-PAGE. Then, proteins were transferred onto PVDF membranes and blotted with 5% non-fat dry milk and corresponding primary antibodies provided in Table S1. After that, the membranes were incubated with appropriate secondary HRP antibodies. Images were captured using chemiluminescent detection system.

### Cell proliferation assay

Cell proliferation was performed using Cell viability Assay Kit (C0068M, Beyotime). GC cells were collected and seeded to 96-well plates at a density of 3000 cells per well. After culturing for the indicated time, CellTiter-Lumi Plus Reagent was added to the cultured medium and incubated for 2 min at room temperature. Then, the luminescent signals were measured with microplate reader.

### Colony formation assay

GC cells were seeded to 6-well plates at a density of 1000 cells per well. After culturing for 2 weeks, colonies were fixed with 4% paraformaldehyde and stained with crystal violet. The number of colonies was counted using a light microscope.

### Cell cycle and apoptosis assays

For determination of cell cycle distribution and percentage of cell apoptosis, a cell cycle assay kit and an AnnexinV/PI apoptosis kit (US Everbright Inc) were respectively used following to their manufacturer’s protocols. Two hours before cell apoptosis analysis, GC cells were treated with an apoptosis inducer CCCP at a concentration of 150 mM.

### Xenograft tumor growth assay

Healthy male BALB/c mice of 4–8 weeks of age were used for the xenograft model experiment. Animal study procedures were approved by the Institutional Animal Care and Use Committee of the Air Force Medical University (Xi’an, China). Briefly, 5 × 10^6^ stable EDDM3A knockdown or control BGC-823 cells were injected into the left flank of each mouse. The mice were divided randomly into EDDM3A knockdown or control group (six mice per group). Tumor volume was measured using a caliper every week for 4 weeks. At the end of the treatment, the animals were sacrificed by asphyxiation with CO_2_, and the tumors were harvested and their weights were measured.

### In vivo metastasis assay

The tail vein metastases model was used for determination of in vivo metastasis ability of GC cells. Briefly, 1 × 10^6^ stable EDDM3A knockdown or control BGC-823 cells were injected into the tail vein of nude mice. The mice were divided randomly into EDDM3A knockdown or control group (six mice per group). The lung tissues were dissected 5 weeks post cells injection and then fixed for H&E staining to quantify metastasis. All the mice experiments were approved by the Institutional Animal Care and Use Committee of the Air Force Medical University (Xi’an, China).

### Generation of 3′-UTR reporter constructs for EDDM3A

The wild-type and mutant 3′UTR sequences of EDDM3A were amplified from the full-length cDNA and subcloned into the pMIR-Reporter vector (Ambion) to obtain 3′-UTR-luciferase reporter plasmid. Mutation of the miRNA seed binding sites was performed using the QuickChange site-directed mutagenesis kit (Agilent). The plasmids were respectively named as EDDM3A 3′UTR-WT and EDDM3A 3′UTR-mut. Each vector along with miR-618 or scrambled control were cotransfected into GC cells using Lipofectamine 3000 reagent (Invitrogen) following the manufacturer’s instructions. Twenty-four hours later, the luciferase activity was measured by Dual-Glo luciferase assay (Promega).

### Glucose uptake and lactate production detection assays

Commercial glucose and lactate detection kits (#F006-1-1, #A019-2-1, Nanjing Jiancheng Bioengineering) were used for detection of glucose uptake and lactate production in GC cells with different EDDM3A levels following to their manufacturer’s protocols. The absorbance was measured using a Bio-Rad microplate reader.

### Evaluations of mitochondrial oxygen consumption and respiratory chain complexes activities

XF96 Extracellular Flux Analyzer (Seahorse Bioscience) was used for determination of the oxygen consumption rate in GC cell lines following the manufacturer’s protocols. For evaluation of the activities of respiratory chain complexes activities, a MitoTox™ Complete OXPHOS Activity Assay Kit (#ab110419, Abcam) was used following the manufacturer’s instruction.

### Measurement of mitochondrial mass

We used a commercially available fluorescent dye MitoTracker Green (Invitrogen, USA) to monitor mitochondrial mass following the manufacturer’s instruction. The results were acquired with a confocal microscope (Olympus, Japan). Fluorescence intensity was assessed by image J software.

### Co-immunoprecipitation (Co-IP) assay

Mix GC cell lysis with protein A beads (Bio-Rad) containing anti-EDDM3A or anti-HIF-1α antibody and rotate the samples at 4 °C for 3 h. The beads were washed three times with cold lysis buffer. The immunoprecipitated samples were finally analyzed by western blot assay.

### Statistical analysis

Experiments were performed in at least three independent replicates. Results are expressed as mean ± standard error of the mean (SEM). Statistical analyses were done using Student’s *t*-tests for two variables or one-way ANOVA analysis for multiple variables. The Spearman rank correlation was used for correlation analysis. Survival analysis was performed using Kaplan–Meier method and log-rank test. *p* value less than 0.05 was considered to indicate statistically significant.

## Results

### EDDM3A expression is increased in GC and inversely associated with patient survival

**Fig. 1:**
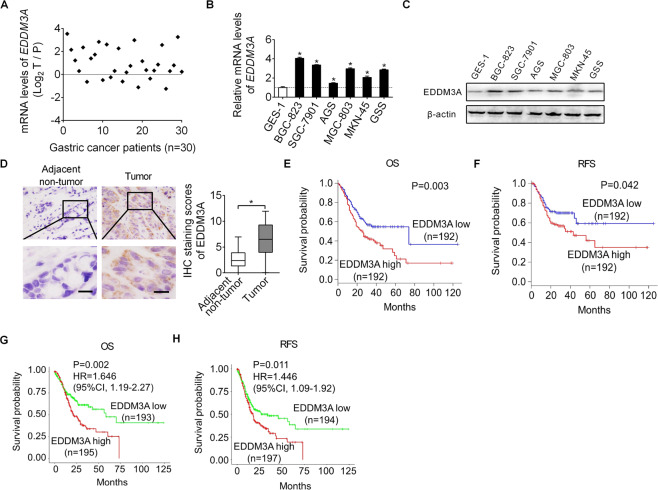
EDDM3A expression is increased in gastric cancer (GC) and inversely associated with patient survival. **A** EDDM3A expression was evaluated by qRT-PCR analysis in 30-paired GC tumor and adjacent non-tumor tissues. **B**, **C** qRT-PCR and western blot analysis for EDDM3A expression in human GC and normal gastric epithelia cell lines. **D** EDDM3A expression was determined by IHC staining analysis at protein level in another 384 paired tumor and non-tumor tissues. Scale bars, 10 μm. **E**, **F** Prognostic significance of EDDM3A expression was analyzed in 384 patients with GC. OS overall survival, RFS recurrence-free survival. **G**, **H** Prognostic significance of EDDM3A expression for GC patients was also analyzed in UALCAN dataset. OS overall survival, RFS recurrence-free survival.

To explore the roles of EDDM3A in GC, we first determined its expression in 30 GC tissues and paired adjacent non-tumor tissues using qRT-PCR analysis. EDDM3A expression was significantly higher in GC tissues than adjacent non-tumor tissues (Fig. 1A). In addition, EDDM3A expression was also significantly elevated in GC cell lines as compared with normal gastric epithelia cell line (Fig. 1B, C). In keeping with these findings, the protein expression of EDDM3A were also found to be significantly higher in tumor tissues of GC than in their adjacent non-tumor tissues, as evidenced by immunohistochemistry (IHC) staining in another 384 paired tumor and non-tumor tissues (Fig. 1D). To estimate the clinical importance of EDDM3A expression in GC, the correlation between EDDM3A expression and the prognosis of patients with GC were analyzed. We found that GC patients with high expression of EDDM3A had significant poorer overall and recurrence-free survival than those with low EDDM3A expression (Fig. 1E, F). Consistently, the prognostic value analysis using LOGpc also showed that GC patients with high EDDM3A expression at mRNA level have significant poor overall and recurrence-free survival than those with low EDDM3A expression (Fig. 1G, H). Moreover, we found that the protein expression of EDDM3A was significantly correlated with the clinicopathological features of tumor size and lymphatic invasion (Table S3). Taken together, the above results indicate that the expression of EDDM3A is significantly increased in GC. EDDM3A may function as an independent prognostic factor for patients with GC.

### Knockdown of EDDM3A suppressed GC growth via inducing cell cycle arrest and apoptosis

**Fig. 2:**
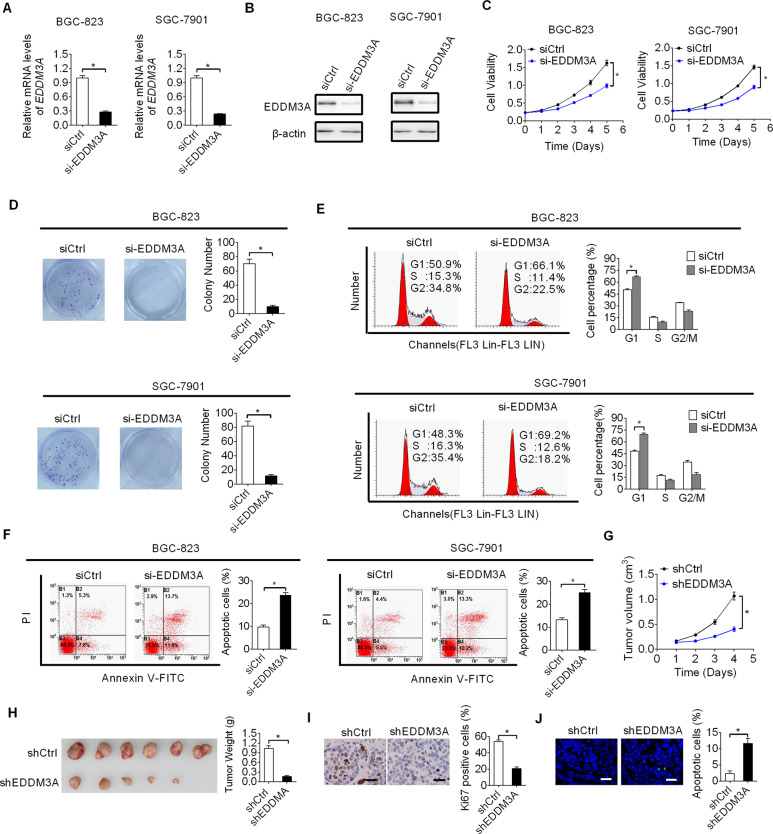
Knockdown of EDDM3A suppressed GC growth via inducing cell cycle arrest and apoptosis. **A**, **B** Knockdown of EDDM3A was detected using qRT-PCR and Western blot analysis in BGC-823 and SGC-7901 cells. **C**, **D** Effects of EDDM3A knockdown on cell viability and colony formation abilities were evaluated in BGC-823 and SGC-7901 cells. **E**, **F** Effects of EDDM3A knockdown on cell cycle progression and apoptosis were determined in BGC-823 and SGC-7901 cells. **G** In vivo tumor growth rates were compared between shEDDM3A and shCtrl groups. **H** Tumor size and weight were compared between shEDDM3A and shCtrl groups. **I**, **J** Ki-67 IHC staining and TUNEL assays were used for detection of cell proliferation and apoptosis in xenograft tumors. Scale bars, 20 μm.

To explore the function of EDDM3A in the tumorigenesis of GC, we transfected small interfering RNA (siRNA) corresponding to EDDM3A into BGC-823 and SGC-7901 cells with relatively high EDDM3A expression to knockdown EDDM3A. Using qRT-PCR and western blot assays, we found that EDDM3A expression was successfully knocked down at both mRNA and protein level in BGC-823 and SGC-7901 cells upon transfecting siRNA targeting EDDM3A (Fig. 2A, B). Next, we investigated the role of EDDM3A in GC growth. Knockdown of EDDM3A resulted in significantly reduced cell viability and colony formation abilities in BGC-823 and SGC-7901 cells (Fig. 2C, D). Cell cycle progression and apoptosis assays using flow cytometry further revealed that knockdown of EDDM3A dramatically induced G1/S cell cycle arrest and increased percentage of cell apoptosis, implying that EDDM3A may promote GC growth through accelerating cell proliferation and reducing cell apoptosis (Fig. 2E, F).

To explore whether EDDM3A regulates tumorigenesis of GC in xenograft tumor model, stable EDDM3A knockdown (shEDDM3A) or control (shCtrl) BGC-823 cells were subcutaneously injected into flanks of nude mice. Significant attenuations of growth rate and tumor weights were observed in shEDDM3A group as compared with those in shCtrl group (Fig. 2G, H). Moreover, in concordance with the in vitro findings, IHC staining of Ki-67 (an indicator of proliferation) and TUNEL staining assay (detection of cells undergoing apoptosis) also showed significantly reduced proliferating and increased apoptotic cells in shEDDM3A tumors as compared with those in control group (Fig. 2I, J).

### Knockdown of EDDM3A attenuated GC cell migration and invasion through suppression of epithelial–mesenchymal transition (EMT)

**Fig. 3:**
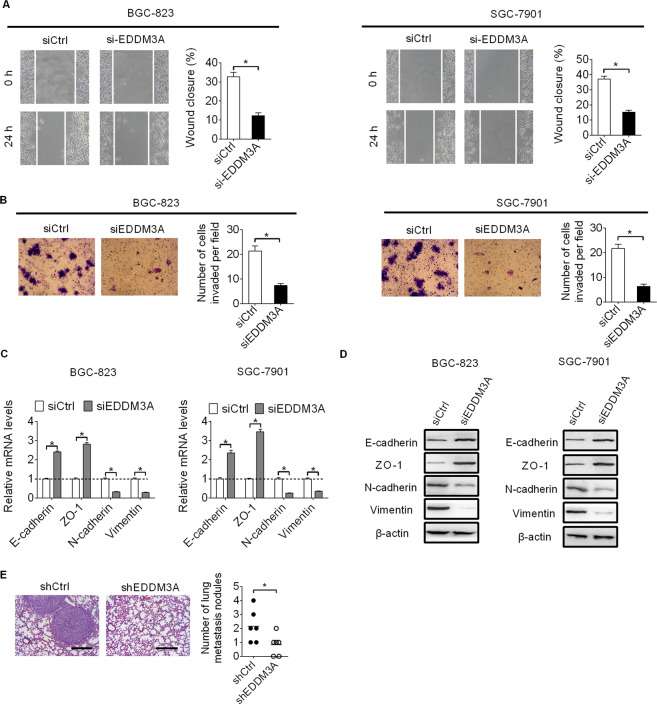
Knockdown of EDDM3A attenuated GC cell migration and invasion through suppression of epithelial–mesenchymal transition (EMT). **A**, **B** Effects of EDDM3A knockdown on cell migration and invasion were evaluated in BGC-823 and SGC-7901 using wound healing and matrigel invasion assays. **C**, **D** qRT-PCR and western blot analysis for the changes of epithelial markers (E-cadherin and ZO-1) and mesenchymal markers (N-cadherin and vimentin) in BGC-823 and SGC-7901 cells, upon EDDM3A knocking down. **E** Metastatic nodules in the lungs from EDDM3A knockdown (shEDDM3A) or control (shCtrl) groups were analyzed using H&E staining assay. Scale bars, 100 μm.

Next, we explored the role of EDDM3A in the migration and invasion of GC cells. Wound healing assay revealed that the wound closure speed was significant slower in EDDM3A knocking down BGC-823 and SGC-7901 cells than in their controls (Fig. 3A). Matrigel invasion assay indicated that knockdown of EDDM3A markedly attenuated cell invasion in BGC-823 and SGC-7901 cells (Fig. 3B). Our next question was whether EDDM3A is involved in the promotion of epithelial-mesenchymal transition (EMT), a key process driving cancer metastasis. Using qRT-PCR and western blot assays, the results showed that knockdown markedly increased epithelial markers (E-cadherin and ZO-1) but decreased mesenchymal markers (N-cadherin and vimentin) in BGC-823 and SGC-7901 cells (Fig. 3C, D), suggesting that knockdown of EDDM3A suppressed epithelial–mesenchymal transition (EMT) in GC cells.

We also explored the effects of EDDM3A knockdown on GC metastasis in vivo by injecting stable EDDM3A knockdown (shEDDM3A) or control (shCtrl) BGC-823 cells into the tail vein of nude mice. The results indicated that knockdown of EDDM3A significantly decreased the number of metastatic nodules formed in the lungs (Fig. 3E).

### Ectopic expression of EDDM3A promoted GC growth and metastasis

**Fig. 4:**
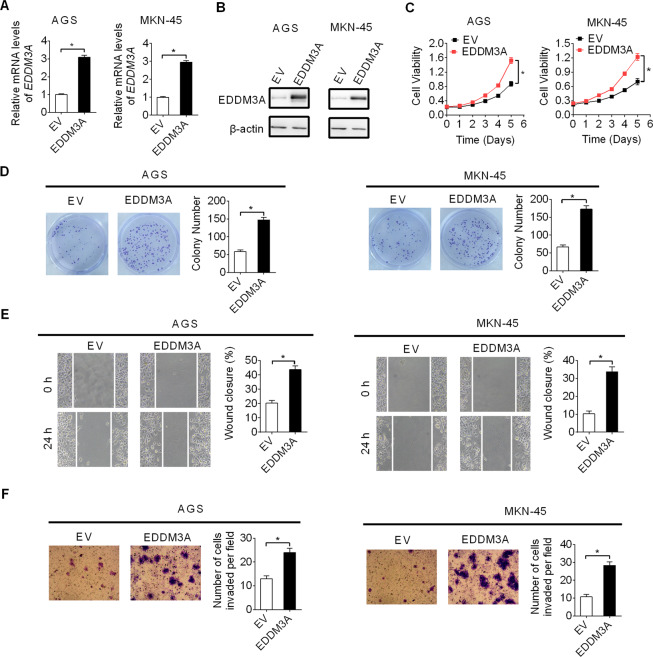
Ectopic expression of EDDM3A promoted GC growth and metastasis. **A**, **B** Forced expression of EDDM3A was confirmed by qRT-PCR and western blot assays in AGS and MKN-45 cells. **C**, **D** Effects of EDDM3A overexpression on cell viability and colony formation abilities were evaluated in AGS and MKN-45 cells. **E**, **F** Effects of EDDM3A overexpression on cell migration and invasion abilities were evaluated in AGS and MKN-45 cells.

To validate the role of EDDM3A in the promotion of growth and metastasis in GC cells, EDDM3A was overexpressed in AGS and MKN-45 cells expressing relatively low EDDM3A by transfecting EDDM3A expression or empty vectors. Successful overexpression of EDDM3A in AGS and MKN-45 cells was confirmed by qRT-PCR and western blot assays (Fig. 4A, B). Overexpression of EDDM3A significantly elevated the viability and colony formation ability in AGS and MKN-45 cells, as demonstrated by MTS cell viability and colony formation assays (Fig. 4C, D). Additionally, overexpression of EDDM3A in AGS and MKN-45 cells also resulted in a remarkable increase of cell migration and invasion, as demonstrated by wound-healing and matrigel invasion assays (Fig. 4E, F). Altogether, the above results further confirm that EDDM3A is a novel oncogene involved in gastric carcinogenesis.

### EDDM3A upregulation is mainly mediated by downregulation of miR-618 in GC

**Fig. 5:**
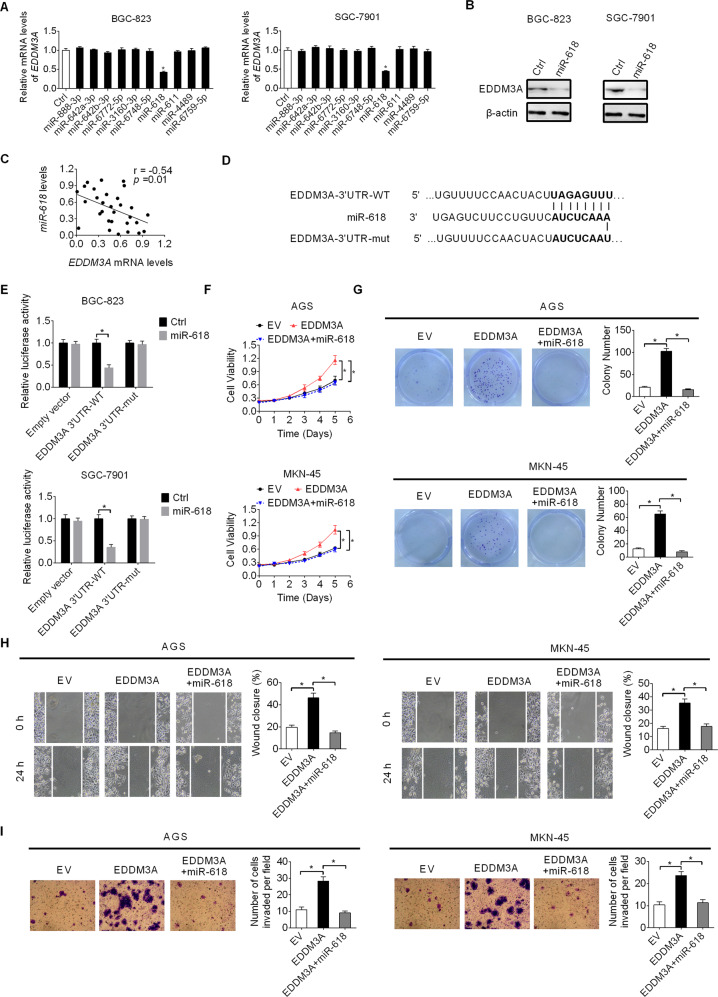
EDDM3A up-regulation is mainly mediated by down-regulation of miR-618 in GC. **A**, **B** The effect of miRNA transfection on the expression of EDDM3A was determined by qRT-PCR and Western blot analysis in BGC-823 and SGC-7901 cells. **C** Correlation between the expressions of miR-618 and EDDM3A in tumor tissues of GC was analyzed (*n* = 30). **D** The binding sites of miR-618 were constructed for wild-type EDDM3A 3′-UTR and the corresponding mutant type. **E** Luciferase assay of EDDM3A 3′-UTR and EDDM3A 3′-UTR-mut reporters cotransfected with miR-618 mimic in BGC-823 and SGC-7901 cells. **F**, **G** Effects of miR-618 transfection on cell viability and colony formation regulated by EDDM3A were determined in AGS and MKN-45 cells. **H**, **I** Effects of miR-618 transfection on cell migration and invasion regulated by EDDM3A were determined in AGS and MKN-45 cells.

MicroRNAs (miRNAs) are critical regulators of genes expression. To investigate whether the upregulation of EDDM3A in GC cells is caused by decreased miRNA expression, potential miRNAs targeting the EDDM3A mRNA were predicted using the microRNA Data Integration Portal (mirDIP) [7]. The results showed that, among the top ten predicted miRNAs targeting EDDM3A (Table S4), the expression of EDDM3A in BGC-823 and SGC-7901 cells was decreased only when transfecting with miR-618 (Fig. 5A, B). In addition, a significant negative correlation was also observed between the expression of miR-618 and EDDM3A in GC tumor tissues from 30 patients (Fig. 5C). To test the binding between miR-618 and the EDDM3A 3′-UTR, a luciferase reporter assay was performed with a wild-type or mutated EDDM3A 3′-UTR coupled luciferase reporter (Fig. 5D). The results showed that the luciferase activity was markedly suppressed by miR-618 in BGC-823 and SGC-7901 cells transfected with wild-type EDDM3A 3′-UTR, while mutation of the 3′-UTR sequence of EDDM3A attenuated the suppression of luciferase reporter activity by miR-618 (Fig. 5E). Moreover, as expected, the promotion of GC cell growth and metastasis by EDDM3A overexpression was also attenuated upon miR-618 transfection in AGS and MKN-45 cells (Fig. 5F–I).

### EDDM3A promotes aerobic glycolysis in GC cells

**Fig. 6:**
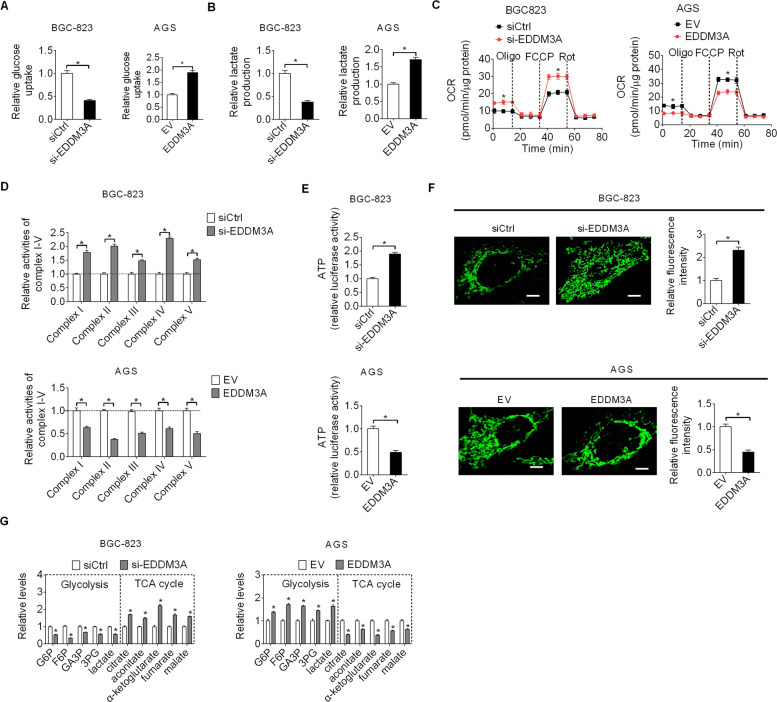
EDDM3A promotes aerobic glycolysis in GC cells. **A**, **B** The effects of EDDM3A knocking down or overexpressing on glucose uptake and lactate production were determined in BGC-823 and AGS cells. **C**–**E** Effects of EDDM3A knocking down or overexpressing on mitochondrial oxygen consumption rate (**C**), OXPHOS complexes activities (**D**), and ATP production (**E**) were determined in BGC-823 and AGS cells. **F** Confocal microscopy assay for mitochondrial mass in BGC-823 and AGS cells with EDDM3A knocking down or overexpressing. Scale bars, 2 μm. **G** The levels of metabolites involved in glycolysis and TCA cycle were determined in BGC-823 and AGS cells with EDDM3A knocking down or overexpressing.

Increased aerobic glycolysis is a common metabolic phenotype that has been extensively observed in a variety of human cancer types, including GC [8]. Notably, we observed that the culture medium turned yellow faster in GC cells with higher EDDM3A expression than that in control cells, implying that EDDM3A may promote acidification by increasing glycolysis. To address this question, the effects of EDDM3A knockdown or overexpression on glucose uptake and lactate production were evaluated in GC cells. The results showed that BGC-823 cells with EDDM3A knocking down displayed a significant reduction of glucose uptake and lactate production as compared with control cells. In contrast, AGS cells with EDDM3A overexpressing exhibited increased glucose uptake and lactate production as compared with control cells (Fig. 6A, B). Considering that a negative feedback is always existing between aerobic glycolysis and mitochondrial oxidative phosphorylation (OXPHOS) [9], we next explored the effects of EDDM3A knockdown or overexpression on mitochondrial oxygen consumption rate (OCR), OXPHOS complexes activities and ATP production. The results revealed that BGC-823 cells with EDDM3A knocking down displayed a significant elevation of OCR (Fig. 6C), activities of OXPHOS complexes I–V (Fig. 6D) and ATP level (Fig. 6E), whereas overexpression of EDDM3A exhibited the opposite effects on these respiratory phenotypes (Fig. 6C–E). Confocal microscopy assay revealed a significant increase in mitochondrial number in BGC-823 cells upon EDDM3A knocking down, while a significant decrease in mitochondrial number in AGS cells upon EDDM3A overexpressing (Fig. 6F), suggesting that EDDM3A may suppress mitochondrial biogenesis in GC cells. These findings were supported by metabolite analysis using gas chromatography-time of flight mass spectrometry (GC-TOF-MS), showing that EDDM3A knockdown decreased the metabolites involved in glycolysis, while increased the metabolites involved in TCA cycle in BGC-823 cells. On the contrary, EDDM3A overexpression in AGS cells exhibited increased metabolites involved in glycolysis, while decreased metabolites involved in TCA cycle (Fig. 6G). Taken together, these results show that EDDM3A promotes aerobic glycolysis in GC cells.

### EDDM3A promotes aerobic glycolysis by upregulating normoxic stabilization of HIF-1α and its target metabolic genes

**Fig. 7:**
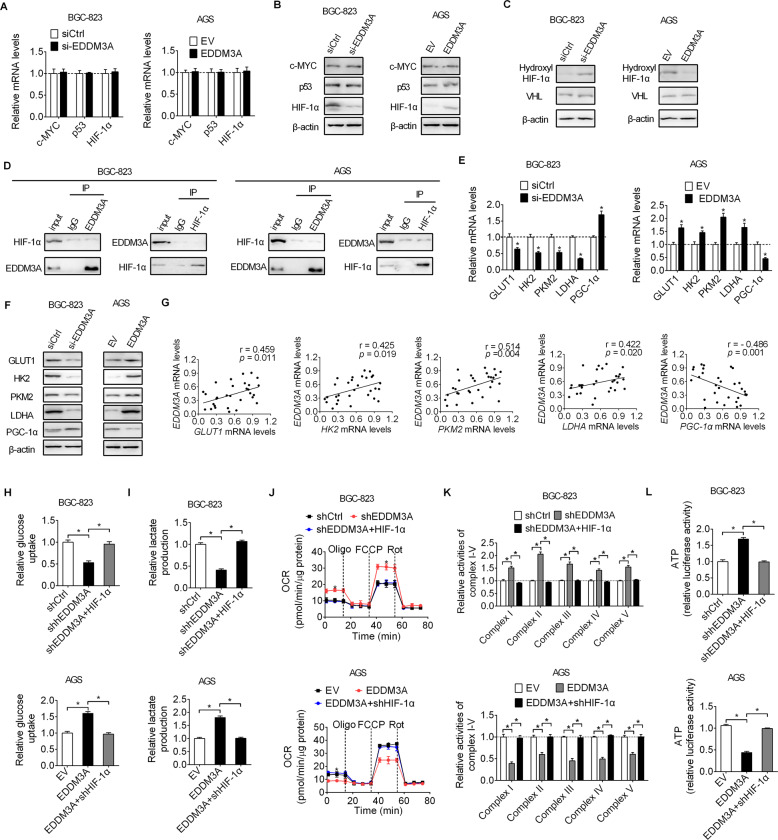
EDDM3A promotes aerobic glycolysis by upregulating normoxic stabilization of HIF-1α and its target metabolic genes. **A**, **B** The effects of EDDM3A knocking down or overexpressing on the expressions of three key transcriptional factors (c-MYC, P53, and HIF-1α) were determined by qRT-PCR and western blot analysis in BGC-823 and AGS cells. **C** The effects of EDDM3A knocking down or overexpressing on hydroxylated HIF-1α protein and VHL expression were determined by Western blot analysis in BGC-823 and AGS cells. **D** Co-IP assay was carried out to explore the interaction between EDDM3A and HIF-1α in the extracts from BGC-823 and AGS cells. **E**, **F** The effects of EDDM3A knocking down or overexpressing on the expressions of glycolytic and mitochondrial biogenesis regulators were determined by qRT-PCR and western blot analysis in BGC-823 and AGS cells. **G** Correlation analysis between the expressions of EDDM3A and the glycolytic genes of GLUT1, HK2, PKM2, and LDH-A, and mitochondrial biogenesis regulator PGC-1α in tumor tissues from 30 GC patients. **H**, **I** Glucose uptake and lactate production were determined in BGC-823 and AGS cells with different treatment as indicated. **J**–**L** Mitochondrial oxygen consumption rate (**J**) and OXPHOS complexes activities (**K**) and ATP production (**L**) were determined in GC cells with different treatment as indicated.

To determine the molecular mechanism by which EDDM3A promotes aerobic glycolysis in GC cells, changes in the expressions of three key transcriptional factors (c-MYC, P53, and HIF-1α) involved in the regulation of aerobic glycolysis were determined by qRT-PCR and western blot analysis upon EDDM3A knocking down or overexpressing. We did not observe any significant changes in the expressions of c-MYC and P53 when EDDM3A was knocked-down or overexpressed, whereas HIF-1α expression at protein level was positively regulated by EDDM3A (Fig. 7A, B). It has been well-known that HIF-1α is hydroxylated and subsequently degraded by the E3 ubiquitin ligase Von Hippel–Lindau (VHL) in normoxia condition [10, 11]. Accordingly, we tested whether EDDM3A upregulated HIF-1α expression through increasing its normoxic stabilization by evaluating the levels of hydroxylated HIF-1α and VHL in BGC-823 cells with EDDM3A knockdown or in AGS cells with EDDM3A overexpression. The results showed that the level of hydroxylated HIF-1α was significantly upregulated or downregulated when EDDM3A was either knocked-down or overexpressed in BGC-823 and AGS cells, while the levels of VHL were not changed following the knockdown or overexpression of EDDM3A in BGC-823 and AGS cells (Fig. 7C), suggesting that EDDM3A upregulated HIF-1α expression in GC cells through increasing normoxic HIF-1α stabilization. We also performed co-immunoprecipitation (Co-IP) assay to explore whether a direct interaction exists between EDDM3A and HIF-1 in GC cells. Unexpectedly, we did not observe any significant direct interaction between EDDM3A and HIF-1 (Fig. 7D), suggesting that HIF-1α is indirectly involved in the role of EDDM3A in gastric cancer cells.

We next explored the role of EDDM3A in the expressions of serials of glycolytic genes. Expectedly, EDDM3A significantly promoted the expression of glycolytic genes including GLUT1, HK2, PKM2, and LDHA, while decreased the expression of mitochondrial biogenesis regulator PGC-1α (Fig. 7E, F). In line with the above findings from cell lines, correlation analysis in tumor tissues from 30 GC patients also indicated significant positive relationships between EDDM3A and the glycolytic genes of GLUT1, HK2, PKM2, and LDHA, while a significant negative correlation between EDDM3A and PGC-1α (Fig. 7G). Moreover, suppressed aerobic glycolysis by EDDM3A knockdown was markedly rescued by overexpression of HIF-1α in BGC-823 cells, while HIF-1α silencing reversed EDDM3A overexpression-induced aerobic glycolysis in AGS cells (Fig. 7H–L). Altogether, these results demonstrate that EDDM3A promotes aerobic glycolysis by upregulating HIF-1α and its target glycolytic genes.

### EDDM3A promoted the growth and metastasis of GC by increasing aerobic glycolysis

**Fig. 8:**
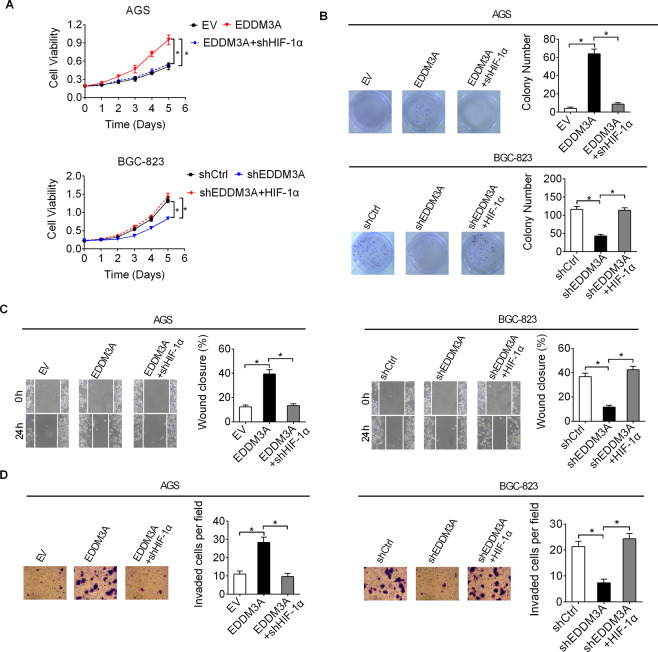
EDDM3A promoted the growth and metastasis of GC by increasing aerobic glycolysis. **A**, **B** In vitro cell proliferation of AGS and BGC-823 cells with different treatment were determined by MTS cell viability and colony formation assays. **C, D** In vitro cell metastasis of AGS and BGC-823 cells with different treatment were determined by wound-healing migration and matrigel invasion assays.

Given that increased aerobic glycolysis plays an important roles in the promotion of tumor growth and metastasis, we therefore tested whether increased glycolysis contributes the promotion of GC growth and metastasis by EDDM3A in GC. As shown in Fig. 8A–D, suppression of glycolysis by HIF-1α knocking down significantly reversed GC growth and metastasis promoted by forced EDDM3A expression in AGS cells, while activation of glycolysis by HIF-1α overexpressing significantly rescued GC growth and metastasis suppressed by EDDM3A knocking down in BGC-823 cells, as determined by in vitro cell proliferation and metastasis assays. These results suggest that EDDM3A overexpression promotes GC growth and metastasis by increasing aerobic glycolysis.

## Discussion

Epididymal protein 3A (EDDM3A) is a protein involved in sperm maturation [5]. A previous study has revealed that EDDM3A is upregulated in non-small cell lung cancer (NSCLC) and promotes NSCLC cell proliferation [6]. However, the role of EDDM3A in other types of human cancers, including gastric cancer (GC), is still unexplored. Here, we demonstrate that the expression of EDDM3A is frequently increased in both tumor tissues and cell lines of GC, which is mainly due to the down-regulation of miR-618. In addition, we found that the upregulation of EDDM3A is closely associated with poor survival for patients with GC. These findings together suggest that EDDM3A plays a crucial role in the development and progression of human cancers.

Significant upregulation of EDDM3A in GC implies that EDDM3A may exert a carcinogenic effect in gastric cancer. Therefore, we investigated the functional roles of EDDM3A in gastric cancer progression by knocking down or overexpression of EDDM3A in GC cells. Knockdown of EDDM3A in BGC-823 and SGC-7901 cells significantly impaired cell proliferation and colony formation. By contrast, forced expression of EDDM3A markedly increased cell proliferation and colony formation abilities in AGS and MKN-45 cells. Consistent with our findings in GC, knockdown of EDDM3A in NSCLC also markedly attenuated the proliferative rate and colony formation abilities in A549 cells [6]. The in vitro results in GC cells was further supported by a xenograft tumor model in nude mice, which showed that the growth rate and weights of tumors developed from BGC-823 cells with EDDM3A stably knocked down were significantly decreased when compared with those developed from the control BGC-823 cells. Considering that increased cell growth could be caused by accelerated cell cycle progression or/and reduced cell apoptosis, we therefore investigated the role of EDDM3A in the regulation of cell cycle progression and apoptosis. Our results indicated that knockdown of EDDM3A dramatically induced G1/S cell cycle arrest and increased percentage of cell apoptosis, suggesting that EDDM3A promotes GC growth through accelerating cell cycle progression and reducing cell apoptosis. In agreement with this, Ki-67 staining and TUNEL assays in xenograft tumors also showed that knockdown of EDDM3A significantly suppressed proliferation but increased apoptosis of GC cells.

We also examined the functional role of EDDM3A in the regulation of metastasis of GC cells. In vitro wound healing and matrigel invasion assays showed that the migration and invasion abilities of BGC-823 and SGC-7901 cells were remarkably suppressed upon EDDM3A knocking down. On the contrary, overexpression of EDDM3A significantly increased the migration and invasion in AGS and MKN-45 cells. We further demonstrated that knockdown of EDDM3A markedly suppressed epithelial–mesenchymal transition, as indicated by increased epithelial markers (E-cadherin and ZO-1) and decreased mesenchymal markers (N-cadherin and vimentin) upon EDDM3A knocking down in. Together, these findings indicate that EDDM3A plays an important role in the promotion of epithelial–mesenchymal transition and metastasis in BGC-823 and SGC-7901 cells.

MicroRNAs (miRNAs) play critical roles in gene expression at post-transcriptional level. MiR-618 has been reported to function as a critical tumor suppressor in several types of human cancers, including prostate cancer [12, 13], osteosarcoma [14], anaplastic thyroid cancer [15]. In gastric cancer, miR-618 has been shown to be downregulated and suppress the metastasis abilities of tumor cells [16]. In addition, it was also reported that miR-618 participated in cisplatin resistance in gastric cancer [17]. Consistent with the above findings in GC, our present study also shows that miR-618 is downregulated in tumor tissues of GC. Moreover, we found that decreased miR-618 expression contributed to the upregulation of EDDM3A and thus promotion of GC cell growth and metastasis. Take into account that gene expression regulation occurs at multiple levels, we do not exclude the possibility that other alterations may also result in the upregulation of EDDM3A in GC cells.

Cancer cells reprogram glucose metabolism from OXPHOS toward glycolysis, even under normoxic conditions, not only to provide sufficient biomass and energy for rapid proliferation, but also generates acid associated with transformation [18, 19]. Characterizing critical factors underlying glycolysis is critical for better understanding of cancer development. In the present study, we demonstrate that the upregulation of EDDM3A plays an important role in glucose metabolism regulation by switching from OXPHOS to glycolysis in GC cells. HIF-1α is a master regulator of glucose metabolism through transcriptional activating a serial of glycolytic genes [20]. Our results showed that EDDM3A promoted aerobic glycolysis through upregulating normoxic stabilization of HIF-1α and its target metabolic genes of Glut1, HK2, PKM2, LDHA, and PGC-1α. It has been well-established that HIF-1α is degraded via the von Hippel-Lindau (VHL)-mediated ubiquitin-proteasome pathway in normoxia condition [20]. Our results indicated that EDDM3A significantly decreased the level of hydroxylated HIF-1α in GC cells, suggesting that EDDM3A upregulates HIF-1α expression in GC cells by increasing normoxic HIF-1α stabilization. In addition, no significant direct interaction between EDDM3A and HIF-1 was observed in GC cells. However, the question regarding the exact mechanisms by which EDDM3A upregulates the normoxic stabilization of HIF-1α in GC cells still needs further exploration.

In summary, we show for the first time that EDDM3A expression is frequently upregulated in gastric cancer cells and its upregulation is closely associated with poor survival of GC patients. EDDM3A plays a critical oncogenic role in the promotion of GC growth and metastasis through enhancing aerobic glycolysis in a HIF-1α-dependent manner. Our findings suggest EDDM3A as a potential prognostic marker and therapeutic target in the treatment of gastric cancer.

## Supplementary information

supplementary tables
