# Supplementary material for: EDDM3A drives gastric cancer progression by promoting HIF-1α-dependent aerobic glycolysis
Source: Oncogenesis. 2022 Jan 17;11(1):3. doi: 10.1038/s41389-022-00379-6 (PMC8764035; doi:10.1038/s41389-022-00379-6)

**Supplemental information**

**EDDM3A drives gastric cancer progression by promoting HIF-1α-dependent aerobic glycolysis**

**Supplemental tables**

**Table S1.** Primary antibodies used in the study.

| **Antibody** | **Company (Cat. No.)** | **Working dilutions** |
| --- | --- | --- |
| EDDM3A | Biorbyt (orb471830) | WB: 1/500; IHC:1/200 |
| E-cadherin | Proteintech (20874-1-AP) | WB: 1/1000 |
| Z0-1 | Proteintech (20742-1-AP) | WB: 1/1000 |
| N-cadherin | Proteintech (22018-1-AP) | WB: 1/1000 |
| Vimentin | Proteintech (10366-1-AP) | WB: 1/1000 |
| MYC | Proteintech (10828-1-AP) | WB: 1/1000 |
| P53 | Proteintech (60283-1-AP) | WB: 1/1000 |
| HIF-1α | Proteintech (20960-1-AP) | WB: 1/1000 |
| hydroxyl HIF-1α | Cell Signaling (NO. 3434) | WB: 1/500 |
| VHL | Santa Cruz (sc-135657) | WB: 1/1000 |
| GLUT1 | Proteintech (21829-1-AP) | WB: 1/1000 |
| HK2 | Proteintech (22029-1-AP) | WB: 1/1000 |
| PKM2 | Proteintech (15822-1-AP) | WB: 1/1000 |
| LDHA | Proteintech (19987-1-AP) | WB: 1/1000 |
| PGC-1α | Proteintech (66369-1-AP) | WB: 1/1000 |
| β-actin | Proteintech (20536-1-AP) | WB: 1/1000 |

**Table S2. Sequence of primers for qRT-PCR analysis**

| **1. Primers used in q-PCR analysis** | | |  |
| --- | --- | --- | --- |
| *EDDM3A* | forward primer | CATTGTGGCGTAGAT GGATA  GGATA | |
|  | reverse primer | ATAAATGTAAGCGGGGAG TG | |
| *E-cadherin* | forward primer | ATTTTTCCCTCGACACCCGAT | |
|  | reverse primer | TCCCAGGCGTAGACCAAGA | |
| *Z0-1* | forward primer | CGACCAGATCCTCAGGGTAA | |
|  | reverse primer | TCCATAGGGAGATTCCTTCTCA | |
| *N-cadherin* | forward primer | TCAGGCTGTGGACATAGAAACC | |
|  | reverse primer | GCTGTAAACGACTCTGGCACT | |
| *Vimentin* | forward primer | GACGCCATCAACACCGAGTT | |
|  | reverse primer | CTTTGTCGTTGGTTAGCTGGT | |
| *miR-618* | forward primer | CGGCGGAAACTCTACTTGTCCTT | |
|  | reverse primer | ATCCAGTGCAGGGTCCGAGG | |
| *U6* | forward primer | CTCGCTTCGGCAGCACA | |
|  | reverse primer | AACGCTTCACGAATTTGCGT | |
| *c-MYC* | forward primer | CTTCTCTCC GTCCTCGGATTCT | |
|  | reverse primer | GAAGGTGATCC AGACTCTGACCTT | |
| *p53* | forward primer | CTGAGGTTGGCTCTGACTGTACCACCATCC TCCTCC-3 | |
|  | reverse primer | CTCATTCAGCTCTCGGAACATCTCGAAGCG | |
| *HIF-1α* | forward primer | CTCCCATACAAGGCAGCAGAAA | |
|  | reverse primer | CAAAACAACCAACAGAAACGAAAC | |
| *GLUT1* | forward primer | CTTTGTGGCCTTCTTTGAAGT | |
|  | reverse primer | CCACACAGTTGCTCCACAT | |
| *HK2* | forward primer | TTGACCAGGAGATTGACATGGG | |
|  | reverse primer | CAACCGCATCAGGACCTCA | |
| *PKM2* | forward primer | ATGTCGAAGCCCCATAGTGAA | |
|  | reverse primer | TGGGTGGTGAATCAATGTCCA | |
| *LDHA* | forward primer | ATGGCAACTCTAAAGGATCAGC | |
|  | reverse primer | CCAACCCCAACAACTGTAATCT | |
| *PGC-1α* | forward primer | CGCACAACTCAGCAAGTCCTC | |
|  | reverse primer | CCTTGCTGGCCTCCAAAGTCTC | |
| *β-actin* | forward primer | TCGCCTTTGCGATCCG | |
|  | reverse primer | ATGATCTGGGTCATCTTCTCG | |

**Table S3. Correlation between EDDM3A expression and clinicopathologic features in 384 patients with gastric cancer.**

| Variables | No. of cases (%) | EDDM3A expression | | *P* value |
| --- | --- | --- | --- | --- |
|  |  | Low | High |  |
| All | 384 (100%) | 192 | 192 |  |
| Age |  |  |  |  |
| <65 | 186 (48.4%) | 91 | 95 | 0.759 |
| >=65 | 198 (51.6%) | 101 | 97 |  |
| Gender |  |  |  |  |
| Female | 106 (27.6%) | 52 | 54 | 0.909 |
| Male | 278 (72.4%) | 140 | 138 |  |
| Differentiation |  |  |  |  |
| High/Median | 172 (44.8%) | 93 | 79 | 0.182 |
| Low | 212 (55.2%) | 99 | 113 |  |
| Lauren’s classification |  |  |  |  |
| Intestinal | 164 (42.7%) | 85 | 79 | 0.606 |
| Diffuse/Mixed | 220 (57.3%) | 107 | 113 |  |
| Size |  |  |  |  |
| <5 | 213 (55.5%) | 118 | 95 | **0.024** |
| >=5 | 171(44.5%) | 74 | 97 |  |
| Lymphatic invasion |  |  |  |  |
| No | 106 (27.6%) | 63 | 43 | **0.030** |
| Yes | 278 (72.4%) | 129 | 149 |  |
| Stage |  |  |  |  |
| I+ II | 175 (45.6%) | 92 | 83 | 0.412 |
| III+ IV | 209(54.4%) | 100 | 109 |  |

**Table S4.** Top ten predicted miRNAs targeting EDDM3A using the microRNA Data Integration Portal (mirDIP).


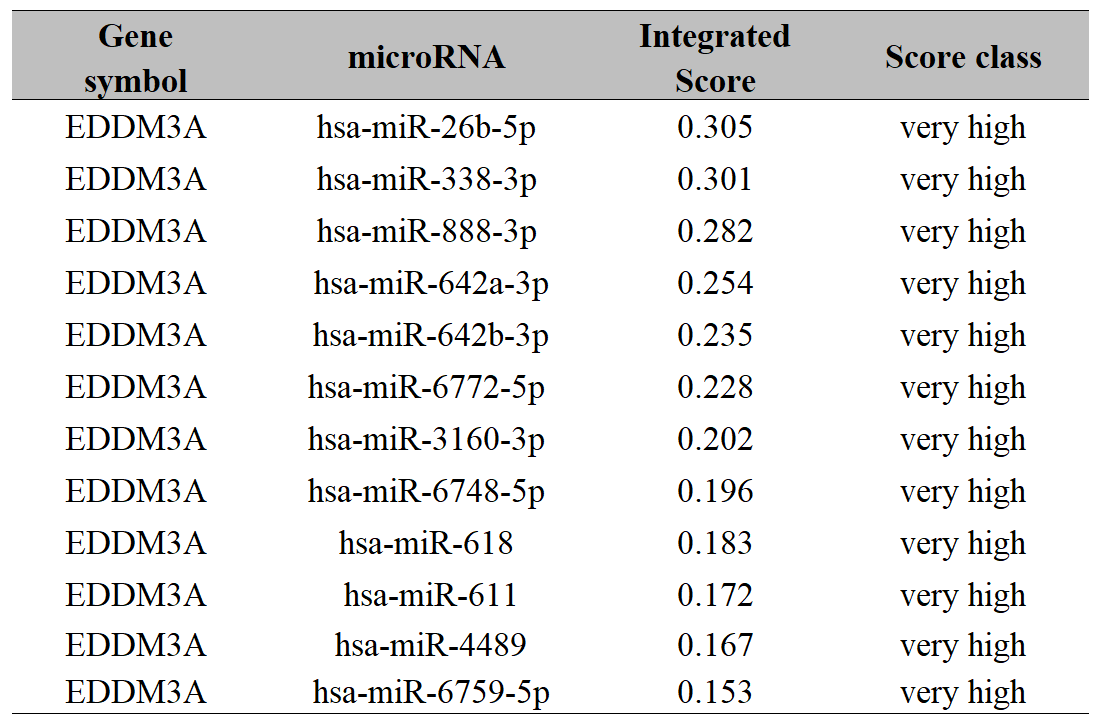

Supplement: Supplementary file 1 — supplementary tables [file 41389_2022_379_MOESM1_ESM.doc]
